# Pore-scale hydrodynamics influence the spatial evolution of bacterial biofilms in a microfluidic porous network

**DOI:** 10.1371/journal.pone.0218316

**Published:** 2019-06-27

**Authors:** Jayde A. Aufrecht, Jason D. Fowlkes, Amber N. Bible, Jennifer Morrell-Falvey, Mitchel J. Doktycz, Scott T. Retterer

**Affiliations:** 1 Biosciences Division, Oak Ridge National Laboratory, Oak Ridge, TN, United States of America; 2 Bredesen Center, University of Tennessee, Knoxville, TN, United States of America; 3 Center for Nanophase Materials Sciences, Oak Ridge National Laboratory, Oak Ridge, TN, United States of America; 4 Biochemistry & Cellular and Molecular Biology, University of Tennessee, Knoxville, TN, United States of America; University of Notre Dame, UNITED STATES

## Abstract

Bacteria occupy heterogeneous environments, attaching and growing within pores in materials, living hosts, and matrices like soil. Systems that permit high-resolution visualization of dynamic bacterial processes within the physical confines of a realistic and tractable porous media environment are rare. Here we use microfluidics to replicate the grain shape and packing density of natural sands in a 2D platform to study the flow-induced spatial evolution of bacterial biofilms underground. We discover that initial bacterial dispersal and grain attachment is influenced by bacterial transport across pore space velocity gradients, a phenomenon otherwise known as rheotaxis. We find that gravity-driven flow conditions activate different bacterial cell-clustering phenotypes depending on the strain’s ability to product extracellular polymeric substances (EPS). A wildtype, biofilm-producing bacteria formed compact, multicellular patches while an EPS-defective mutant displayed a linked-cell phenotype in the presence of flow. These phenotypes subsequently influenced the overall spatial distribution of cells across the porous media network as colonies grew and altered the fluid dynamics of their microenvironment.

## Introduction

Microbial communities are complex systems that shape, and are shaped by, their local microenvironments. Bacteria often inhabit heterogeneous microenvironments with hydrodynamic flows that influence local nutrient transport and chemical gradients, creating specialized niches for microorganisms[1–3]. Likewise, the spatial confinement of some bacteria can influence emergent phenomena like quorum sensing, intercellular communication, and biofilm formation[4–6]. These microenvironment factors and complexities contribute to microbial community diversity and synergism, which hinders their isolation and culture in bulk laboratory conditions[7,8].

Soil exemplifies a complex and heterogeneous microbial environment. Within soil, the physical and chemical structure of the porous network dictates water and nutrient flow, influencing the cells’ spatial distribution, communal behaviour, evolution, and even cross-kingdom interactions[9–11]. Some bacterial species can produce extracellular polymeric substances (EPS), which improve a soil’s moisture retention and act to aggregate soil grains together, further altering underground hydrodynamics[12,13]. Thus, bacterial characteristics are tightly coupled with the dynamics of soil conditions. This bacteria-soil interplay has implications for bioremediation, water quality, nutrient cycling, and underground ecology. It is therefore necessary to study soil bacteria within the structural and hydrodynamic context of their natural environment and on length scales appropriate to cellular functions (i.e. the pore scale) to elicit emergent behaviours. Unfortunately, the opacity of soil presents a challenge for the direct visualization and measurement of bacterial traits at the pore-scale *in situ*.

Experiments in sand columns have enabled measurements of bulk bacterial transport through porous media and have even allowed some preliminary imaging of bacteria in pore-spaces[14–17]. Nafion, a transparent fluoropolymer that is sometimes used as a sand substitute, can further increase the imaging compatibility of bacteria in flow cells[18,19]. However, these systems do not have a defined structure and are often treated as ‘black boxes’, making it impossible to correlate pore-scale hydrodynamics with bacterial biofilm distribution.

In other synthetic systems, microfluidic platforms have been used to visualize bacterial behaviour in flow through narrow channels and around tight corners[20–22]. These platforms reduce the physicochemical complexity of natural porous media while testing bacterial characteristics in highly parameterized and fully defined systems[23]. Microfluidic systems have the added benefit of retaining the same physical structure for each experimental replicate, allowing flow in the channels to be computationally simulated[24,25]. These systems have elucidated bacterial chemotaxis through tortuous channels, bacterial streamer formation, and microbial competition[9,26]. However, microfluidic platforms have not yet been employed to study how the coupling of flow and biofilm ability affect the spatial distribution of bacteria in heterogeneous porous media.

Microfluidic platforms can also be designed with increasing complexity to approximate the porous media structure of natural environmental systems. Many researchers have used homogeneous designs of circular features with varied packing densities, radii, and pitches to approximate porous structure [27–30]. While the reduction of grain shape to equivalent spheres has been successfully used in the field of soil physics to describe averaged fluid space quantities such as porosity and permeability, the size of a single bacterial cell is on a length scale well below the representative pore space volume that justifies the spherical grain assumption[31,32]. In order to inform individual based models of soil microbial community behaviours, an accurate grain shape is needed to reproduce the fluid dynamics and labyrinthine pore spaces that an individual microbe would experience underground.

Some microfluidic designs have taken the heterogeneity of natural porous media into consideration. Pore throat networks inspired by reservoir rock have been reconstructed through the use of Voronoi cell tessellations[33], and focused ion beam-scanning electron microscopy (FIB-SEM) image triangulation, but geometrically partitioning the pore space results in a loss of information from the original sample[33,34]. Another team used an algorithm generator to create ellipsoid-shaped grains with a pore space reminiscent of natural granular media and, within their platform, demonstrated the influence of EPS on underground hydration processes[35]. The ellipsoidal shapes of the generated porous media, however, are not based on shape statistics collected from natural grains.

In this work, we replicate both the two-dimensional shape and packing distributions of natural sand in a 2D microfluidic platform. A reduction in dimension from natural 3D soil substrates, while retaining the porosity of 3D soils, enables a quick image acquisition time to capture the dynamics of bacteria-grain interactions. Our heterogeneous design retains the pore-scale physical structure of natural porous media while providing a fully defined, tractable model for characterizing pore space hydrodynamics. Using gravity fed flow, we characterize the spatial evolution of bacterial biofilms in the porous network and correlate biofilm attachment and growth to pore scale hydrodynamics. We find that rheotaxis, or directed bacterial movement across a velocity gradient, has the biggest influence on the initial spatial distribution of bacteria, but over time EPS production ability and biofilm expansion ultimately determines the spatial distribution of bacteria in this heterogeneous network.

## Results and discussion

### Device design and simulation of pore space hydrodynamics

**Fig 1:**
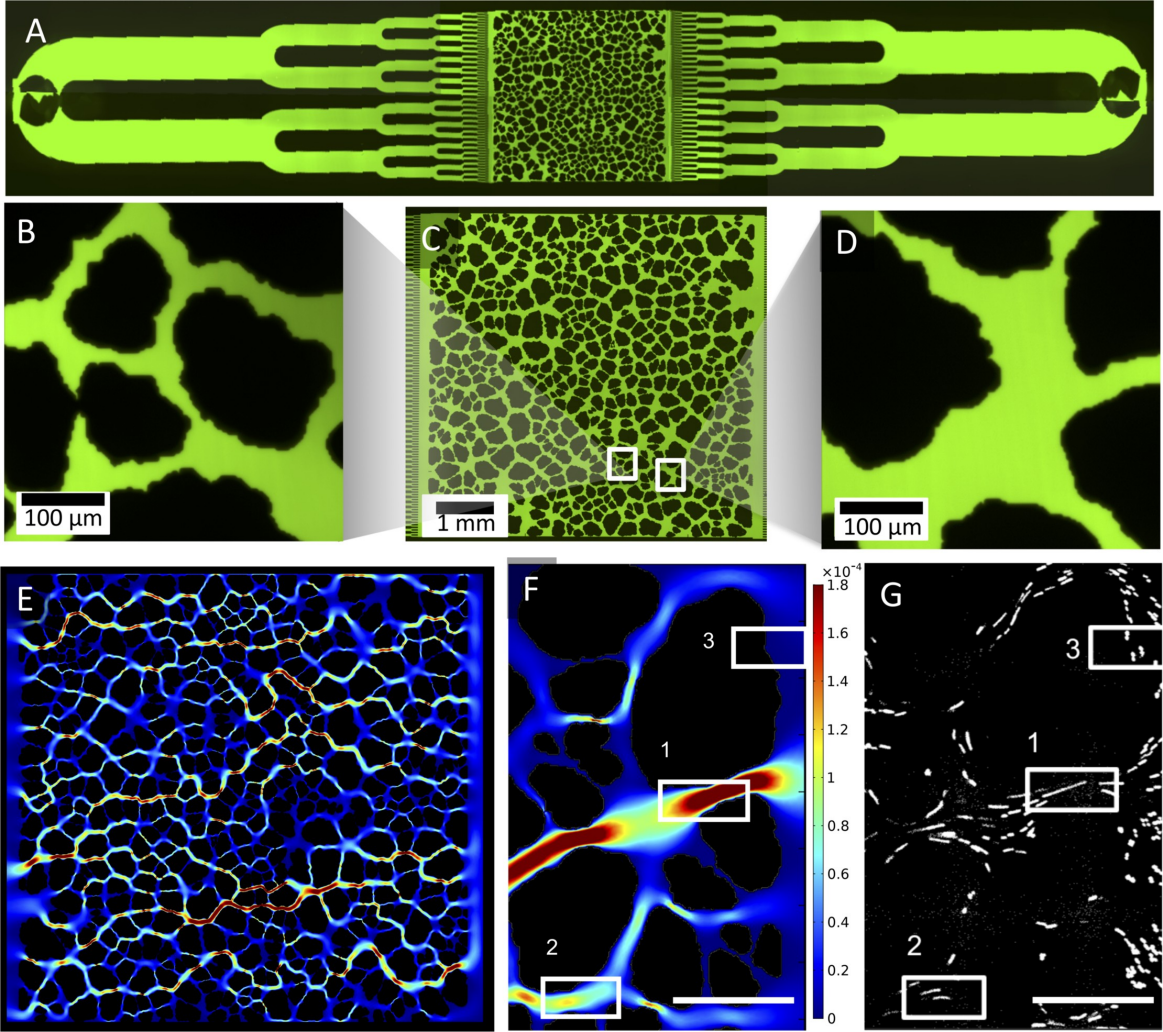
A microfluidic platform replicates the natural shape and layout of sand grains. (A) The device, filled with fluorescein to highlight its features, has a bifurcating inlet and outlet to uniformly distribute bacterial cells across the design (C) The porous network design (approximately 6,000 x 6,000 x 10 um) consists of heterogeneous pore spaces that have (B) constriction points smaller than the size of a bacteria cell and (D) larger, highly connected pore spaces. (E) Velocities within the pore space were simulated using COMSOL Multiphysics, which illuminated multiple preferential flow paths across the system. (F,G) The pore space velocities were experimentally verified using particle image velocimetry and velocity magnitudes (F, regions 1, 2, 3, decreasing in magnitude) corresponded to bead speeds within the same pore (G, regions 1, 2, and 3)(scale bars = 100 μm, velocity units = m/s).

The microfluidic grain design was created using a granular media algorithm to randomly generate 500 grains from the size and shape distributions of natural sands[36,37]. Sand was chosen as a physical model for soil, the latter consisting of variable amounts of sand, silt, clay, and humus that form micro- and macro-aggregate structures. The resulting porous media section of the microfluidic platform contains a heterogeneous network of pore sizes and shapes including closed junctions between grains that can strain cells to large pores with high connectivity (Fig 1A–1D). The distribution of pore spaces closely resembles a lognormal function with a median width of 50μm (S1 Fig). A large variety of pore feature allows for many sub-experiments on biofilm growth in various hydrodynamic conditions to be conducted at once.

The microfluidic porous media maintains the same grain layout and characteristics for each experimental replicate, which provides a highly tractable method to correlate pore scale features with flow. Here the CAD file used to fabricate the microfluidic platform also created the geometry of a COMSOL Multiphysics laminar flow simulation to determine the hydrodynamic flow parameters at steady state for constant velocity inputs. A velocity magnitude heat map was generated to visualize flow between pores and illuminated several preferential flow paths across the system (Fig 1E). To confirm the accuracy of the COMSOL model, particle image velocimetry was used to calculate average flow speeds within pores, which matched the velocity magnitudes simulated in the COMSOL model (Fig 1F and 1G).

### Bacteria growth and flow rate are coupled in a pressure driven system

To recreate the flow of percolating rainwater, a reservoir was suspended above the microfluidic platform and connected by tubing, generating gravity-driven flow. Prior to the experiment, either a wild type (WT) or EPS defective mutant (ΔUDP) of *Pantoea* sp. YR343, a rod-shaped, motile soil bacterium isolated from the rhizosphere of *Populus deltoides*, was grown overnight, diluted, and grown again to mid-exponential phase at an optical density (at 600nm) of 0.1 (OD_600_ = 0.1)[38]. The bacteria were then loaded into the reservoir and seeded into the device for one hour. After one hour, the reservoir was exchanged for sterile R2A media, keeping a constant hydrostatic pressure, and the tubing was also replaced to prevent bacterial growth upstream of the device. The device was then imaged every hour for 21 hours. Additional tubing at the outlet carried effluent from the microfluidic platform to dish on a scale where it was weighed every 10 minutes to monitor the flow rate through the platform (S3 Fig). Prefilling and sealing the dish minimized weight variations due to evaporation.

**Fig 2:**
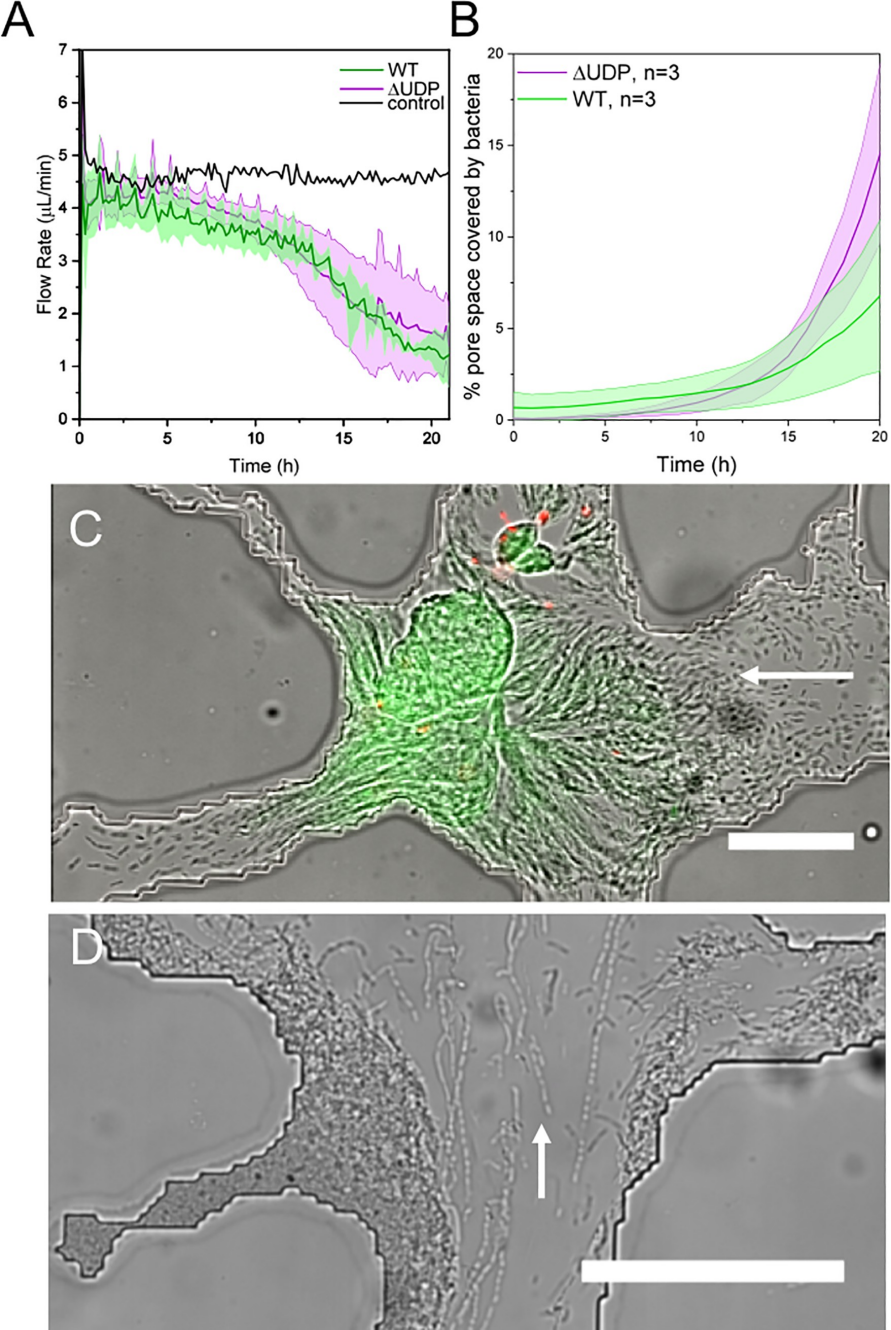
A WT strain and an EPS defective mutant (ΔUDP) grow within the heterogeneous porous media platform. A) Both strains cut off flow at the same rate (n = 4, average values shown by dark line while error (standard deviation) is filled with a lighter color) B) After 21 hours, the ΔUDP strain covers the same area of the device as the wildtype strain. (n = 4, average values shown by dark line while error (standard deviation) is filled with a lighter color) C) Flow (direction indicated by white arrows) induces a globular phenotype for the WT strain (live cells (green) and dead cells (red) are overlaid on the DIC image), and a D) linked-cell phenotype for the ΔUDP strain. (scale bars = 100 μm).

Suspending the reservoir 32 cm above the platform resulted in a constant control flow rate of approximately 5 μL/min. After cells were seeded in the platform, this flow rate steadily dropped off over time, approaching 1 μL/min for both strains after 21 hours (Fig 2A). Despite a reduced capacity to create EPS, a precursor to biofilm development, the ΔUDP strain showed no significant difference in flow rate change compared to the WT strain. However, change in flow rate over time in this heterogeneous porous network was drastically different than the abrupt, catastrophic clogging that other researchers have seen from similar experiments in a single tortuous channel[26]. Presumably, this is because, as bacteria obstruct one preferential flow path, other preferential flow paths open up and divert flow around the clog. This diversion of flow, or remodeling of the porous network, would have environmental implications for chemical transport in natural porous media. Other researchers have found that, as bacteria grow to clog pore spaces, flow redirects to provide nutrients to slower growing colonies[9]. This work suggests that slow growers have the advantage in heterogeneous porous networks because they maintain a sustainable growth rate without cutting off their own nutrient supply.

The ΔUDP mutant cells also did not show a significant difference in pore space coverage by the end of the experiment compared to the WT strain (two-sample t-test, p>0.05) (Fig 2B). Given that the EPS defective strain cuts off flow at the same rate as the WT and grows to cover a similar area, the heterogeneous structure of the porous media does not seem to have interacted with bacterial genotype under these experimental conditions to produce different growth results. However, this system did not test bacterial competition, antibiotic susceptibility, or challenges to the community that would favor a robust biofilm producing strain[39].

Each bacterial strain exhibited unique multi-cellular phenotypes within the microfluidic pore space compared to static bulk media laboratory conditions (i.e. agar plate assays). In the pore network, the WT strain formed compact, globular patches of living cells with a diameter approximating the size of the pore in which the bacteria were confined (Fig 2C). The ΔUDP mutant exhibited phenotypes that depended upon flow within the pore space. In pores with flow the ΔUDP cells formed long chains of linked cells, while cells in static pores retained a single cell, rod-shaped structure (Fig 2D). This ΔUDP phenotype was an important precursor for pore space clogging *in lieu* of EPS formation; strands of living and dead cells spanned multiple grains, forming a foundation on which more cells could attach and grow. It is unclear whether the flow-induced phenotypic changes in this study are purely a passive result of physical forces, or if the cells are sensing local hydrodynamic changes and regulating gene expression accordingly. There is evidence that at least some bacteria are able to transduce mechanical signals into the biochemical signals needed to promote cell attachment to surfaces and initiate biofilm formation [40]. Increasing fluid shear has also been shown to increase the expression of genes responsible for virulence in enterohemorrhagic *E*. *coli* [41]. However, the multicellular phenotypes exhibited by these bacteria may also be a result of non-uniform nutrient transportation (due to pore clogging) or, in the case of stagnant pores, the local accumulation of microbial signaling molecules.

**Fig 3:**
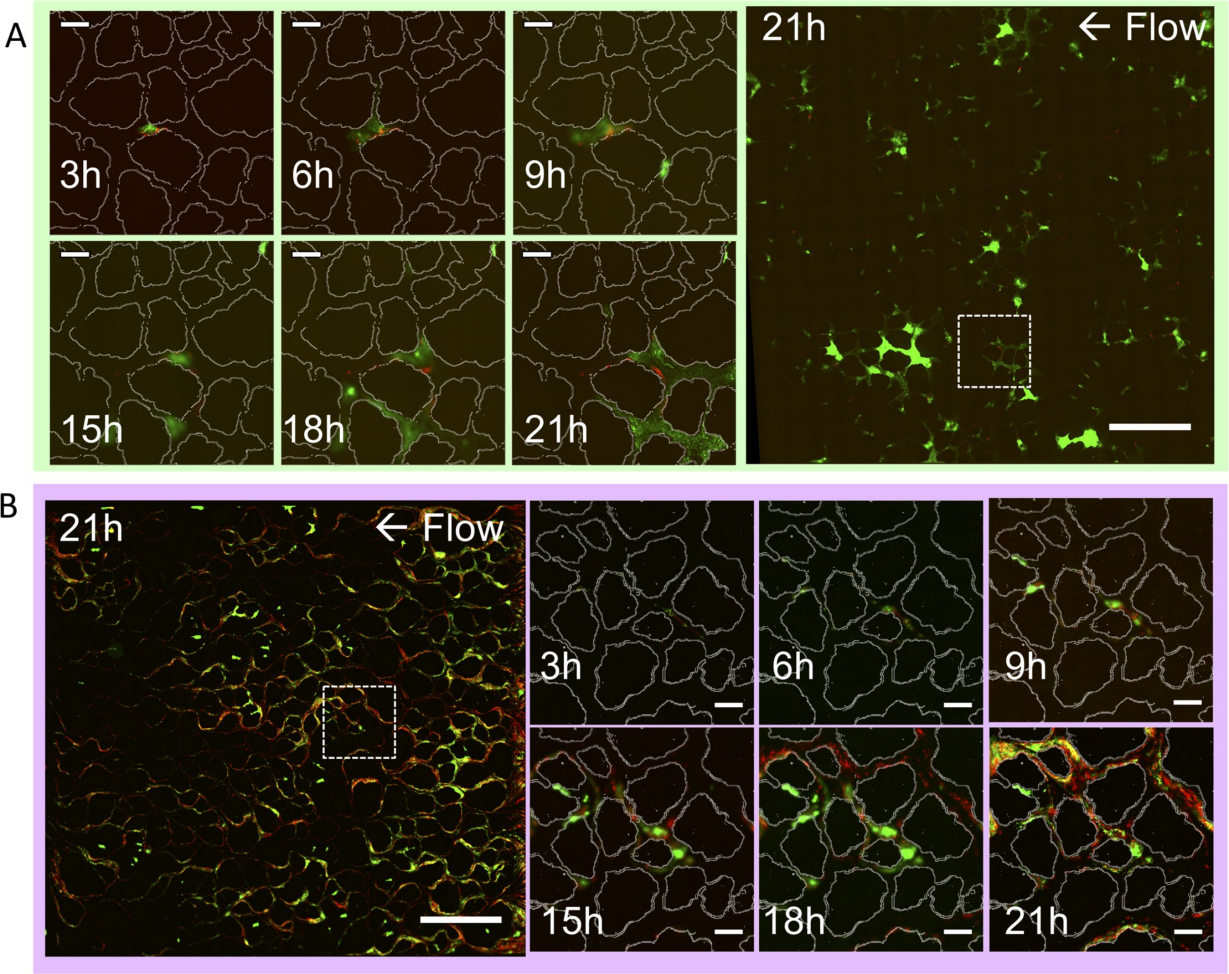
Representative fluorescent images show *Pantoea* cells growing within the pore space over time. A) WT and B) ΔUDP demonstrate different spatial distribution of living (green) and dead (red) cells across platform after 21 hours (scale bars = 1mm). Images from a subset (indicated by dashed box) of the device show biofilm growth within a pore over time with the grain design overlaid (white) (scale bars = 100 μm).

Although the ΔUDP mutant and wildtype bacteria exhibited similar influence over the overall flow through the porous media platform, their pore-scale growth restructured the network flow profiles differently. The linked-cell phenotype of the ΔUDP mutant manifested itself across the pore network in a streamer-like distribution of cells that follows the general direction of flow (Fig 3B). The WT strain, however, exhibited a much patchier distribution of growing microcolonies across the entire pore space (Fig 3A). A patchy distribution of soil organisms is traditionally thought to be a direct result of heterogeneous resource allocation[3, 42]. In this system, however, nutrients are universally dispersed in the media and continually replenished.

The spatial distribution of both bacteria strains across the porous network after 21 hours appeared to evolve from the location of the initially attached bacteria (Fig 3). Therefore, we next examined the simulated hydrodynamics within the pores where we saw the highest incidence of initial attachment, or seeding.

### Correlating initial bacterial attachment with flow hydrodynamics

**Fig 4:**
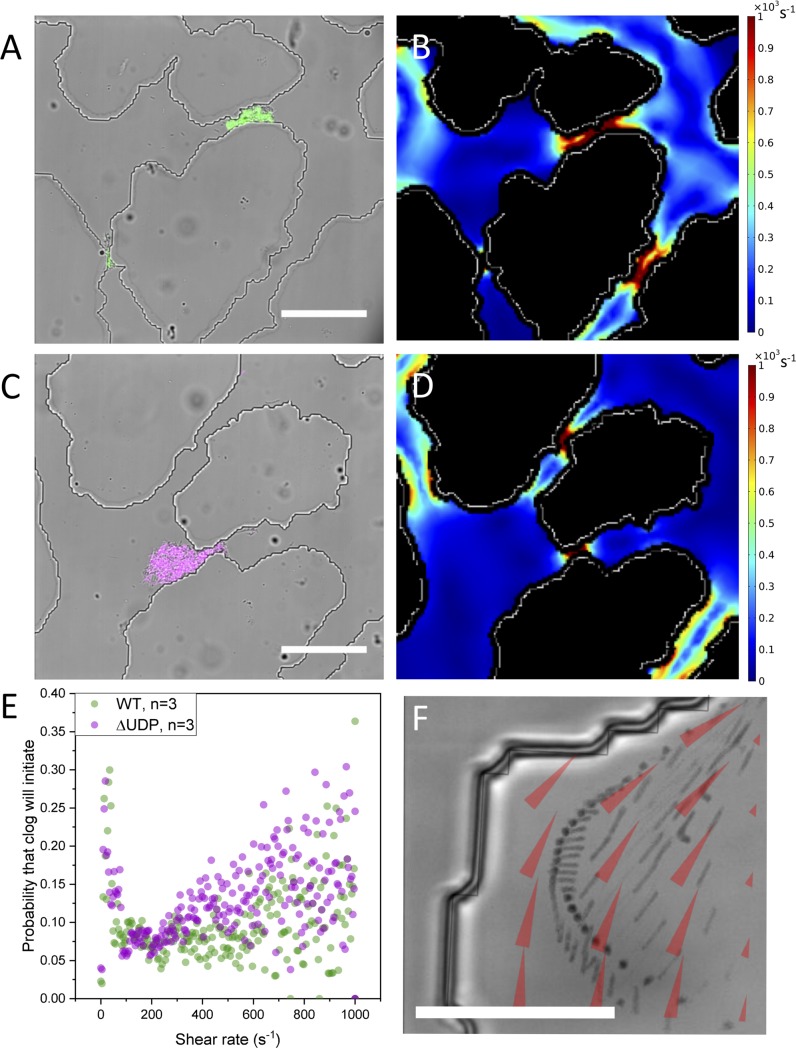
Pore-scale flow interacts with EPS ability to create different cellular phenotypes. Differential interference contrast (DIC) images from representative experiments overlaid with falsely-coloured fluorescent images of A) WT and C) ΔUDP cells show that the location of initial bacteria seeding correlate to (B,C) areas of high simulated shear from the COMSOL simulations (scale bars = 100 μm). E) The probability of a clog occurring in a pore is high approaching a simulated shear rate of zero, and increases again with simulated shear rate. F) A minimum intensity projection over time of WT bacteria cells (black) moving across the velocity gradient (overlaid red arrows from COMSOL simulation) confirming rheotaxis in the pore spaces. (scale bar = 25 μm).

Despite the reproducibility of the pore space layout between experiments, bacterial fluorescence was not always located in the same pores after one hour of seeding, neither between bacterial treatments (WT or ΔUDP) nor between replicates (n = 3). While we attempted to control the initial conditions as much as possible, this result may be, in part, due to minor experimental variations that amplified across the network [43]. Although bacteria did not always initially clog the same pores between replicate experiments, there was a trend in the hydrodynamics of pores where cells were initially captured. In comparing the simulated hydrodynamic conditions of the pores where cells were captured, the probability of clog initiation was highest in two shear regimes: in pores with very low simulated shear rates (approaching 0 s^-1^) due to uniform velocity profiles across the pore space, or at the maximum simulated shear rate in the device (1000 s^-1^)(Fig 4).

Because shear rate is the differential of velocity, extremely low shear rates (approaching 0 s^-1^) can occur whenever there is constant velocity across a pore. However, because of the no-slip boundary condition (zero velocity) at the grain surfaces, the only pores with constant velocity will be the pores with static fluid conditions. The clogging maximum in the pores with extremely low simulated shear could therefore be occurring because the velocities within these pores are low enough for *Pantoea* sp. YR343 swimming speeds (mean swimming speed: 3.5 μm/s, max swimming speed: 7 μm/s) to overcome. Bacterial motility is therefore likely to be the dominant transport mechanism in these pores.

Aside from the pores with approximately zero simulated shear, the probability of clogging steadily increased with shear rate up to the maximum simulated shear rate in the device, 1000 s^-1^. At these high shear rates cells are directed toward grain surfaces by a transport mechanism known as rheotaxis [44]. In bacteria, rheotaxis is passively directed movement across velocity gradients due to lift forces on the bacteria flagella and drag forces on the bacteria cell body[45]. Because velocity at the surface of a grain is zero, following the no-slip boundary condition, high shear rates tend to occur close to grain surfaces thus encouraging cell-grain interactions and, subsequently, biofilm initiation. Videography of cell trajectories confirmed that rheotaxis was directing cells across velocity gradients toward grain surfaces (Fig 4F). This mechanism of microbial transport through soil is a significant departure from colloid filtration theory (CFT), which is sometimes used to estimate the transport of bacteria cells underground, because rheotaxis cannot act on spherical, non-motile colloids.[45–47]

We wanted to understand the relationship between shear rate, pore size, and initial bacterial clogging within the network. An algorithm was created (see S1 Method) to quantify the average width of each pore within the microfluidic platform (S1 Fig). The subset of pores that initially captured WT cells (from three replicate experiments) had a significantly different size distribution than the platform’s overall pore size distribution (paired t-test, two tails, bin size 10μm, p<0.05) with the clogged pores being much smaller on average (mean width = 20 μm).

Each pore’s average width was also compared to the average simulated shear rate within the same pore. Theoretically, if two pores of the same size were aligned in series, the shear rate (and subsequent pore clogging) would be the same for both pores, following the Hagen-Poiseuille equation [48]. However, because the pores experience different flow rates across the network, pore size was not strongly correlated to the simulated shear rate across the entire platform. Pores with the same size had different simulated shear rates depending on the pore’s location with respect to preferential velocity flow paths throughout the network. Therefore in porous media networks, transport due to rheotaxis cannot be predicted solely from pore size.

In addition to being an important factor in trapping bacteria during the initial stages of biofilm seeding within the pore space, we next tried to elucidate the role of rheotaxis in shaping growing biofilms over time.

### Predicting the spatial evolution of bacterial biofilms

Once bacteria have attached to the surface of a grain, their distribution is affected not only by flow, but also by growth[49,50]. Daughter cells may detach and be carried downstream to attach in another location. They can also reattach upstream when cells have specialized characteristics like curved structure or twitching motility[51,52]. Under these growth conditions, the distribution of live cells and pore scale hydrodynamics are coupled. As resistance in the system builds and overcomes the pressure at the device inlet, pore flow decreases locally and bacteria can use swimming motility as a dominant transport mechanism.

To determine if shear trapping via rheotaxis was dictating the spatial distribution of cells during the growth phase, we converted images of living cells in the device at an initial time (t_i_) to CAD files and incorporated them into the geometry of the COMSOL simulation (see Materials and Methods, S2 Fig). The simulated shear profile was then recalculated and compared to the distribution of living cells one hour later (t_i+1_) using object overlay analysis. The probability of co-localization between cells at t_i+1_ and shear simulated from t_i_ was relatively constant for i = 3, 9, and 15 hours indicating that shear trapping is no longer a dominate mechanism determining the spatial distribution of a growing biofilm. However, this method of modeling biofilms is limited and might be improved by adding polymeric material properties to the biofilm geometry and incorporating a solid-liquid interaction into the simulation.

**Fig 5:**
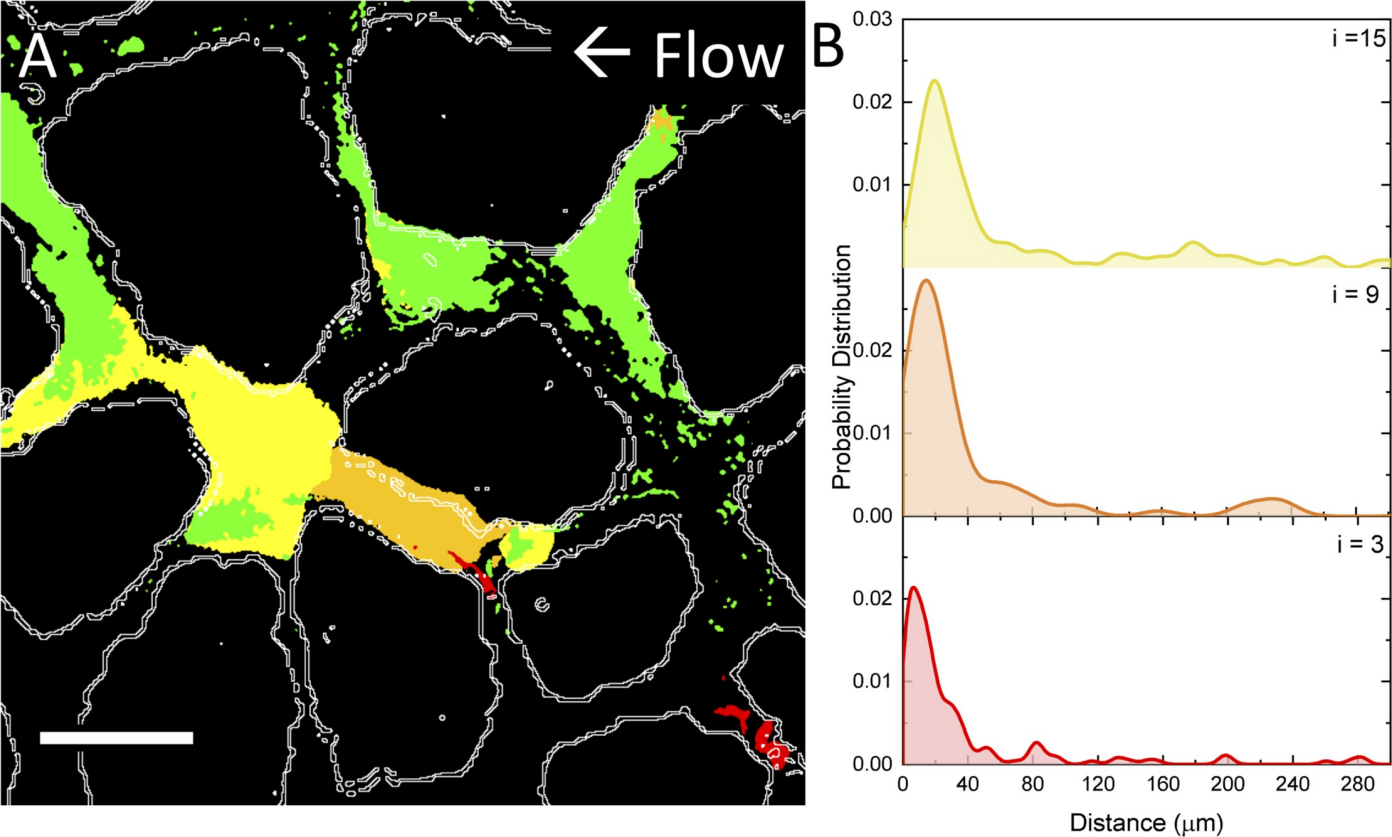
The spatial distribution of biofilms over time depends on the historical location of the mother cell. (A) The change in WT bacterial fluorescence over time from 0–3 hours (red), 3–9 hours (orange), 9–15 hours (yellow), and 15–21 hours (green) show that bacteria biofilms are growing steadily in a downstream direction. The original fluorescent images were thresholded, subtracted, and falsely colored to highlight the changes to the biofilm over time. (B) The nearest neighbour probability for i = 3, 9, and 15 hours shows that nearest neighbors are within a 20 μm radius (not considering pore space tortuosity).

Next we conducted a nearest neighbor interaction analysis for the wildtype bacteria to understand how the spatial distribution of bacterial biofilms changed over time. The bacterial fluorescence was compared in pairs of images (t_i_ and t_(i+1)-i_ for i = 3, 9, and 15 hours) using a nearest neighbor interaction analysis plugin in Fiji [53,54]. The probability of a cell cluster encountering a neighboring cluster peaked at the relatively close distance of 6, 15, and 20 μm for growing cells at i = 3, 9, 15 hours, respectively (Fig 5). By looking at the change in bacterial fluorescence over time, changes indicate that the biofilms grew through pore spaces in a downstream direction. This result suggests that the spatial distribution of the growing biofilm over time depends largely on the historical location of the mother cells which, in turn, demonstrates how early events (i.e. seeding location) can amplify into large changes in flow and biofilm development across the pore network. However, other factors may also be contributing to the changes in fluorescence besides growth such as cell decay and biofilm dispersal.

Other authors have also determined through nearest neighbor analysis that most soil bacteria interactions occur within a 20μm radius[10]. If the fluid dynamics of this system were static, this result would suggest that bacteria interactions are largely clonal, occurring mostly between descendants of the same ancestor cell. However, flow acts to disperse cells, metabolites, and signaling molecules to downstream microcolonies, increasing the extent of bacterial interactions, albeit in one direction. Therefore, biofilm growth and EPS production, although jeopardizing the incoming flow of nutrients by choking off flow, may provide a mechanism for increasing local communication and bi-directional exchange (e.g. horizontal gene transfer) between bacterial microcolonies in the soil.

This work demonstrates the value of studying microbes in complex systems like porous media in order to accurately capture natural microbial characteristics and community interactions. Here, the use of a faithfully replicated physical environment reveals that microbial dispersal and initial attachment in porous media is strongly influenced by shear forces within pore spaces. Results from this work (i.e. cell phenotype changes, spatial distribution, and rheotactic transport) testify to the ability of heterogeneous matrices to influence emergent microbial properties compared to bulk laboratory methods, and these results can be used to validate individual based biofilm models. While this platform only replicates the 2D physical structure of natural sands, increasing complexity can be incorporated into the design, including chemical treatments and nutrient gradients, to recreate natural microbial habitats and emergent bacterial phenomena in a fully defined, parameterized approach.

## Methods

### Device design and fabrication

Using a published granular media algorithm with defined input parameters (S1 Table), the porous media design was created to replicate the shape distribution of sand grains from Tecate, Mexico[36,37]. The algorithm generated a .bmp file of the grain layout that was vectorized in LayoutEditor to create the CAD design. The grain size of the CAD file, defined by the D_50_ gradation, was scaled to 0.13 mm to reflect smaller sand grains. The design’s porosity (0.38), defined as the ratio of open space to solid space, approximated the porosity of sandy loam (0.25–0.35) [35]. A bifurcating inlet and outlet were added to the CAD to uniformly distribute bacteria across the design. The smallest inlet and outlet dimensions were 22 μm in width. This size was chosen in order to uniformly deliver bacterial cells across the porous media platform, as any bias in bacterial introduction would amplify through the entire network.

The design was transferred onto a chrome mask using a Heidelberg DWL 66 mask writer and replicated onto a silicon wafer using standard photolithography techniques with an NFR photoresist and Bosch etch (Oxford RIE) for to give a final depth of 10μm. This depth was chosen to confine bacteria to a single focal plane during the course of the experiment. Trichloro(1H,1H,2H,2H-perfluoro-n-octyl)silane), 85°C, 60 min) was evaporated onto the surface of the silicon wafer in order to prevent adhesion during the polymer molding process. The microfluidic devices were replicated from the silicon master using poly-dimethylsiloxane (PDMS; 5:1 PDMS base to curing agent, wt/wt; Sylgard 184, Dow Corning) and cured overnight at 70°C [55]. Inlet and outlet holes were created using a 1.5 mm biopsy punch and the PDMS was bonded to a glass coverslip using air plasma treatment.

### Bacteria growth

*Pantoea sp*. YR343, a plant growth promoting rhizosphere isolate from the *Populus deltoides* microbiome, was chosen for its relevance to soil ecology and for its rod-shape and flagellar motility[38,56,57]. Fluorescent strains were constructed by integrating GFP into the chromosome of Pantoea sp. YR343 using the pBT270 and pBT277 plasmids as previously described[38]. The EPS mutant was isolated by screening a Pantoea sp. YR343 transposon library on Congo Red plates. The transposon insertion site for the EPS-defective mutant was confirmed to disrupt gene PMI39_01848, which is located at the beginning of an operon with homology to EPS biosynthesis gene clusters found in Pantoea stewartii and Erwinia amylovora[58].

Fluorescence varieties of the WT and ΔUDP strains were grown overnight prior to the experiment on liquid R2A media (TEKnova, Inc.) at 37°C. Bacteria cultures were diluted 100x in fresh R2A the next morning and allowed to grow again to mid exponential phase at an optical density (600nm) of 0.1, as measured by UV spectroscopy.

### Experimental setup

Large (for media) and small (for seeding bacteria) reservoirs were created by piercing a hole at the bottom of 50mL and 15mL centrifuge tubes and securing a 23 gauge blunt-tip needle to the tube with polyurethane glue (DUCO cement). Inlet and outlet Tygon tubing was measured to 25cm and 44cm, respectively. The microfluidic device was placed under vacuum for one minute to facilitate pre-filling with R2A media. Once filled, the device, inlet and outlet tubing, and the reservoirs were UV sterilized for 15 minutes in a UV Stratolinker oven. The materials were assembled on the microscope stage before the start of the experiment as shown in S3 Fig.

A bacteria culture (either WT or ΔUDP strain) was loaded into the smaller reservoir so that the top of the liquid was 32cm above the microfluidic platform inlet. This ensured a consistent pressure-driven flow at 3136 Pa following the hydrostatic pressure head equation (P = ρgh) assuming that the cultures have the same density (ρ) as water. The tubing was attached to the microfluidic inlet and the cells were seeded into the device for 1 hour, after which the reservoir was exchanged for the larger R2A media reservoir taking care to keep the same hydrostatic pressure. The tubing connecting the reservoir to the platform was also changed at this time. Propidium iodide (PI) (0.025μM) was added to the R2A reservoir to stain dead cells over time.

Effluent from the microfluidic platform was carried to a Petri dish on an analytical scale. The dish was sealed with Parafilm to limit evaporation and pre-filled with water to quickly establish a steady-state vapor pressure in the dish. The scale was imaged every 10 minutes and the flow rate was calculated with the assumption that the liquid had the same density of water.

After seeding, bacteria within the microfluidic platform were imaged every hour using an inverted microscope (Nikon Ti-Eclipse) with FITC and TRITC epifluorescence to capture both living and dead cells. Image acquisition was carried out with a 20x objective using Nikon Elements software and a back-illuminated iXonEM+ 897 camera (Andor).

### COMSOL simulation

The porous media CAD was incorporated as the pore space geometry in a 2D laminar flow (spf) COMSOL Multiphysics (version 5.2) simulation. The fluid space was assigned the material properties of water while the grain space was subtracted from the geometry. One side of the design was assigned as an inlet boundary with an entrance pressure of 313.6 Pa (3,136 Pa calculated from the hydrostatic pressure divided by 10 μm for the device height) and the other side of the design was assigned an outlet pressure condition of zero Pa. Due to anisotrophy in grain layout, care was taken during experimental setup to match the experimental inlet with the simulated inlet. An extra-course free triangular mesh was used to partition the geometry into finite elements. The laminar flow study used a stationary GMRES solver (50 iterations) with a relative tolerance of 0.001.

An outlet flow rate from the simulation was calculated by multiplying the line integrated velocity magnitude at the outlet by the device depth (10 μm). This value (4.706μL/min) closely matched the experimental control flow rate of only R2A media of 4.48μL/min (n = 1). Experimental particle image velocimetry using fluorescent 2μm carboxylated polystyrene beads confirmed that the simulated velocity matched experimental average speeds in the pore spaces.

For flow simulations with the biofilms, a fluorescent image of both live and dead cells at a given time (t_i_) was converted to a CAD file (.dxf) and subtracted from the COMSOL geometry. This method worked reasonable well at earlier time points (i = 3, simulated flow rate = 4.56μL/min and experimental flow rate = 4.64μL/min) but the experimental and simulated flow rates diverged at i = 9 (simulated = 4.57μL/min, experimental = 3.91 μL/min). This method of modeling biofilms, is therefore limited, and might be improved by adding polymeric material properties to the biofilm geometry and incorporating a solid-liquid interaction into the simulation. However, the rheological properties of this particular bacterial biofilm remain uncharacterized.

### Image analysis

To remove out-of-focus autofluorescence from debris and background noise, images were processed using Fiji[59]. For each image, the background fluorescence was removed (sliding parabloid, 700μm) and then images were manually thresholded to further reduce noise. Total fluorescence area was analyzed using the particle analyzer feature.

#### Object overlay analyses

For object overlay analyses with the COMSOL simulations, experimental images were rotated, cropped, and downscaled to align with the same region of interest in the COMSOL shear rate profiles. A composite image was created from the experimental and COMSOL images using the AND Boolean operation. The histogram of greyscale pixel values from this composite image provided information on the shear rate values that were co-localized with each bacterial fluorescence pixel. To obtain the “probability that a clog will initiate” metric for each shear rate value, the shear rate associated with each bacterial fluorescence pixel at i = 1 was normalized by the total number of pixels from the COMSOL simulation with the same shear rate value.

#### Nearest neighbour interaction analyses

Change in bacterial fluorescence was calculated by subtracting an image at an initial time (t_i_) from an image one hour later (t_i+1_). The resulting image (t_(i+1)-i_) was compared to t_i_ using the Mosaic Interaction Analysis Plugin to determine nearest neighbours for i = 3,9, and 15[54].

### Pore space characterization

A pore characterization program was created as an addendum to the 2D granular space simulation (GSS) reported by Mollon and Zhao[37]. The purpose of the complementary pore characterization program was to quantify the average width of each pore generated using the GSS. Pores are represented as linear segments in the GSS and the length of the linear segment is defined here as (L). The blue linear network shown in S1 Fig. (top panel) represents the Voronoi tessellation. An iterative algorithm is required to converge to an approximation of the real tortuous pore pathlength. The pore width is ultimately derived from this pore pathway reconstruction. S1 Fig. shows in the initial distribution of transverse pore lines in red (**-**) while the final transverse profile representing an approximation of the tortuous pore pathlength is shown by the population of black lines (**-**). Details of the pore characterization program can be found in the S1 Method.

## Supporting information

S1 Method A description of the algorithm used to characterize the statistical attributes of each pore within this design. (PDF)

S1 Fig (top left) Pore characterization program results shown in a 200 pixel × 200 pixel region–of–interest sampled from a larger 2D granular space of roughly 2000 pixels × 2000 pixels. Pores in the tessellation pattern with total path length less than 10 pixels were not included in the pore characterization statistics. (top right) The subset of clogged pores has a significantly different size distribution than the overall pore size distribution (paired t-test, two tails, bin size 10**μ**m, p < .05). (bottom left) A histogram of the pore widths shows a close resemblance to a lognormal function. (bottom right) Every pore is plotted by its average shear rate and width.
(TIF)

S2 Fig **(**left) An example of the simulated shear rate before (t_0_) and after subtracting the biofilm from the geometry (t_i = 3_) shows that biofilm influences pore space shear rates. (right) The relationship between shear rate and the probability of a clog occurring is relatively constant over time.
(TIF)

S3 Fig A schematic illustrating the experimental setup and the calculation of hydrostatic pressure. The lid on the reservoir tube was loosely fitted to assure atmospheric pressure above the liquid.
(TIF)

S1 Table Input parameters for the grain generating algorithm[37]. (TIF)
