# Supplementary material for: Pore-scale hydrodynamics influence the spatial evolution of bacterial biofilms in a microfluidic porous network
Source: PLoS One. 2019 Jun 27;14(6):e0218316. doi: 10.1371/journal.pone.0218316 (PMC6597062; doi:10.1371/journal.pone.0218316)
Supplement: S1 Method — (PDF) [file pone.0218316.s001.pdf]

Supplementary Methods

Pore characterization program

Purpose

The pore characterization program was created as an addendum to the 2D granular space simulation (GSS) reported by Mollon and Zhao[37]. The purpose of the complementary pore characterization program is to quantify the statistical attributes of each pore generated using the GSS. Pores are represented as linear segments in the GSS and result from a Voronoi tessellation[37]. The length of the linear segment is defined here as (L). The blue and linear network shown in figure S1a represents the Voronoi tessellation. Additional pore attributes of interest that are not provided by the GSS include the average pore width, the variation of the pore width and an approximation of the actual tortuous pathlength between neighboring grains. The pore characterization program is now summarized.

Pore Definition

The pore characterization program is described from the point–of–view of a single pore for clarity. The linear segment defining the pore, derived in the GSS, is sub–divided into multiple, equally spaced nodes (M). The total number of sub–division nodes (M) is an input parameter and ultimately controls the spatial sampling resolution of the pore. Each individual sub–division node is referenced using (m). The user also provides a parameter, range 0–1, that represents the fraction of segment ignored during segment sub–division, defined as (f). The purpose of this parameter is to avoid sampling the pore width at the ends of the segment where the pore is poorly defined in space, as described next.

Multiple pores converge at a common node due to the Voronoi tessellation used to create the granular space using GSS. In combination with the GSS cell filling procedure, these nodal areas tend to produce relatively large open spaces at pore entry and exit surfaces. During the sub–division procedure in the pore characterization program, nodes introduced near the segment ends sample these open spaces, instead of the pore space, which leads to an overestimate of both the mean pore width and mean pore width variation. To avoid this effect, the parameter (f) was introduced to prevent nodal points near the segment ends. This is accomplished by leaving a linear space equal to (f*L/2) free of sub–division nodes at the pore entry and exit. Quantitatively, this results in the following nodal range of points (**P**);

$$\boldsymbol{P} = \frac{f}{2}L : \frac{(1-f)}{M-1}L : \left(1-\frac{f}{2}\right)L \quad [1]$$

where **P** is defined along the linear segment. The origin is assigned arbitrarily and can be either segment node. In this work, the linear segment node with the smallest (x) value is taken as the origin. This arbitrary designation was made simply because the general fluid

flow direction during experiments is oriented along (+x). The pore is therefore initially defined by the vector;

$$\vec{s} = (x_M - x_1)\vec{\imath} + (y_M - y_1)\vec{\jmath} \quad [2]$$

The distribution of initial nodes **P** are indicated with open red circle data points (**o**) in figure S1a.

Pore Shape Extraction

An iterative algorithm is required to converge to an approximation of the real tortuous pore pathlength. The pore width is ultimately derived from this pore pathway reconstruction. *Convergence is obtained when the following two conditions are met for each sub–division node along the pore; (1) the center position (x,y) of the pore node (m) has been found and (2) this center position (x,y) also intersects the line spanning the pore which best aligns with the surface normal on each side of the pore*. The user defines the number of iterations to convergence. Figure S1a shows the outcome of an acceptable level of convergence for pore definition using the pore characterization program. Specifically, the black data points (**x**) show the final distribution of pore sub–division nodes following convergence. A summary of the iterative algorithm is now provided.

The Iterative Solver

The initial condition is a series of equally spaced nodes lying on the linear segment defining the pore (*see previous section*). The normal vectors, oriented orthogonal to the initial pore vector $\vec{s}$, are calculated for each node defined in the range **P**. This calculation requires that the binary, digital image of the granular space map be superimposed with the pore network defined by the Voronoi tessellation. Ultimately, this overlay is necessary for determining the pore width at each sub–division node. In the binary digital image, a pixel value of 0 represents occupied granular space while a pixel value of 1 signifies open 'pore' space. The pore width per node (m) is calculated in the following way.

First, the normal vector per node is derived from the local tangent vector to the current pore path estimate. *Importantly, this tangent vector is calculated using the two nearest neighbor nodes as*;

$$\vec{t}_m = t_{x,m}\vec{\imath} + t_{y,m}\vec{\jmath} = (x_{m+1} - x_{m-1})\vec{\imath} + (y_{m+1} - y_{m-1})\vec{\jmath} \quad [3]$$

where the index (m) represents the current node of interest. By using the 1st nearest neighbor nodes, the solution propagates in an interconnected way through the pore giving a smooth estimate of the tortuous pore pathlength at convergence. The normal vector $\vec{n}$ is derived using;

$$n_{x,m} = -t_{y,m} \quad [4a]$$

$$\vec{\boldsymbol{n}}_m \cdot \vec{\boldsymbol{t}}_m = 0 \quad [4b]$$

yielding the components $n_{x,m}$ and $n_{y,m}$.

Next, a line is cast through the node (m) aligned along $\vec{\boldsymbol{n}}_m$. An estimate of the pore cross–section at (m) is measured as the displacement (□x, □y) between the two points of intersection between the extended line and the neighboring grains forming the pore. Please note, the exact path of the line through each pixel is measured. This minimizes the error incurred when deriving a linear path measurement from a pixelized image to a value of roughly twice the pixel edge length. The current node (m) position (x_m,y_m) is then updated as the average value of the two intersection points. This summary represents the complete sequence of events during a single iteration. The process repeats until convergence is reached. Figure S1a shows in the initial distribution of transverse pore lines in red (-) while the final transverse profile representing an approximation of the tortuous pore pathlength is shown by the population of black lines (-). For Figure S1a, the number of convergence iterations was set to 40, the number of sub–division nodes per pore was M = 13, and the fraction of total pore length ignored during sampling was f = 0.34. Pores in the tessellation pattern with a total pathlength less than 10 pixels were not included in the pore characterization statistics.

Generated Data

Following successful program execution, a text file is saved that contains the following information for each sub–division per pore (m); the pore width, the pore node position and the normal vector defining the pore cross–section at (m). Moreover, this data is provided for each iteration. This information is also provided for all pores in the 2D granular space.

Additional Features

Lastly, it is worth briefly mentioning additional features of the program. A program component was included which prevents pores on the simulation boundary from contributing to the final pore characterization statistics. Also, an additional tool is included making it possible to ignore ‘pores’ that are too short in pathlength to represent an actual pore but were nonetheless generated as part of the Voronoi tessellation procedure. These ‘imposter’ pores were typically created when a relatively large number of pores converged at a single vertex in the Voronoi tessellation pattern.
