# Supplementary figures and images for: Pore-scale hydrodynamics influence the spatial evolution of bacterial biofilms in a microfluidic porous network
Source: PLoS One. 2019 Jun 27;14(6):e0218316. doi: 10.1371/journal.pone.0218316 (PMC6597062; doi:10.1371/journal.pone.0218316)

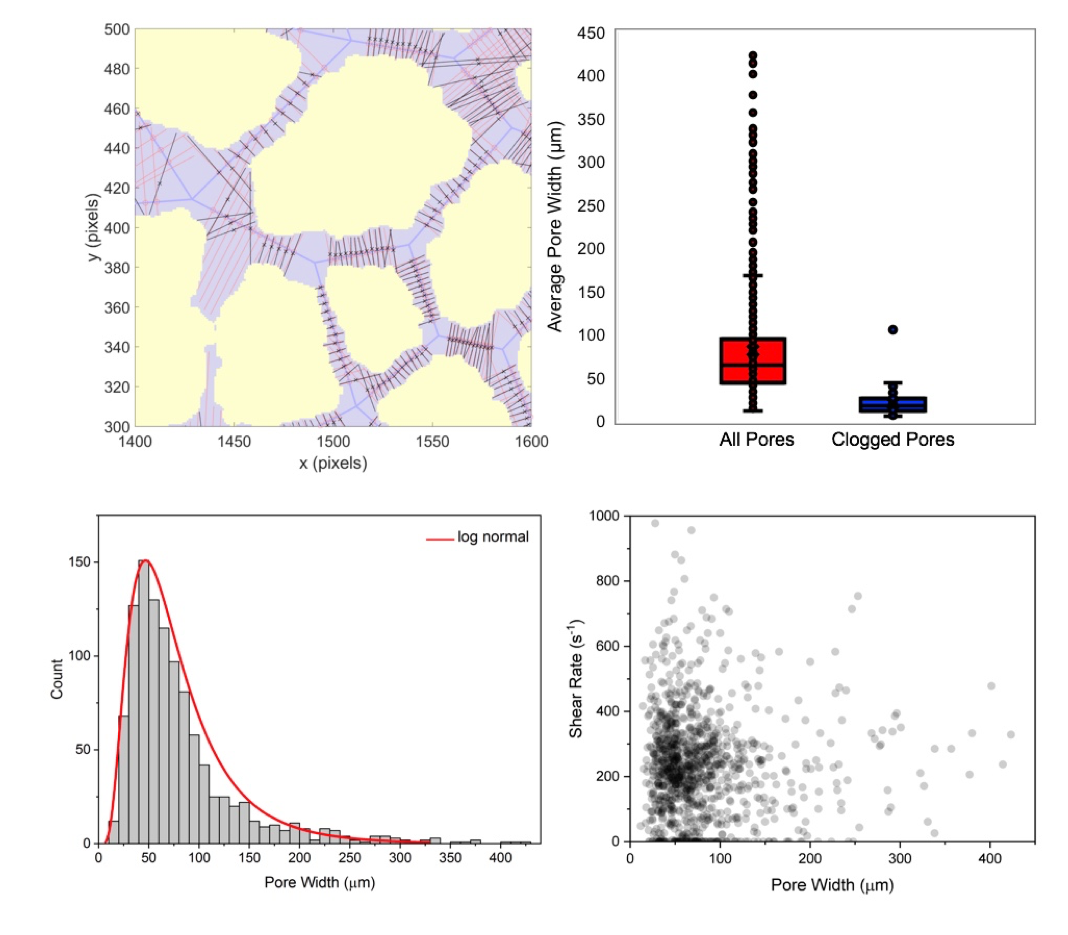

Supplement: S1 Fig — (top left) Pore characterization program results shown in a 200 pixel × 200 pixel region–of–interest sampled from a larger 2D granular space of roughly 2000 pixels × 2000 pixels. Pores in the tessellation pattern with total path length less than 10 pixels were not included in the pore characterization statistics. (top right) The subset of clogged pores has a significantly different size distribution than the overall pore size distribution (paired t-test, two tails, bin size 10μm, p < .05). (bottom left) A histogram of the pore widths shows a close resemblance to a lognormal function. (bottom right) Every pore is plotted by its average shear rate and width. (TIF) [file pone.0218316.s002.tif]

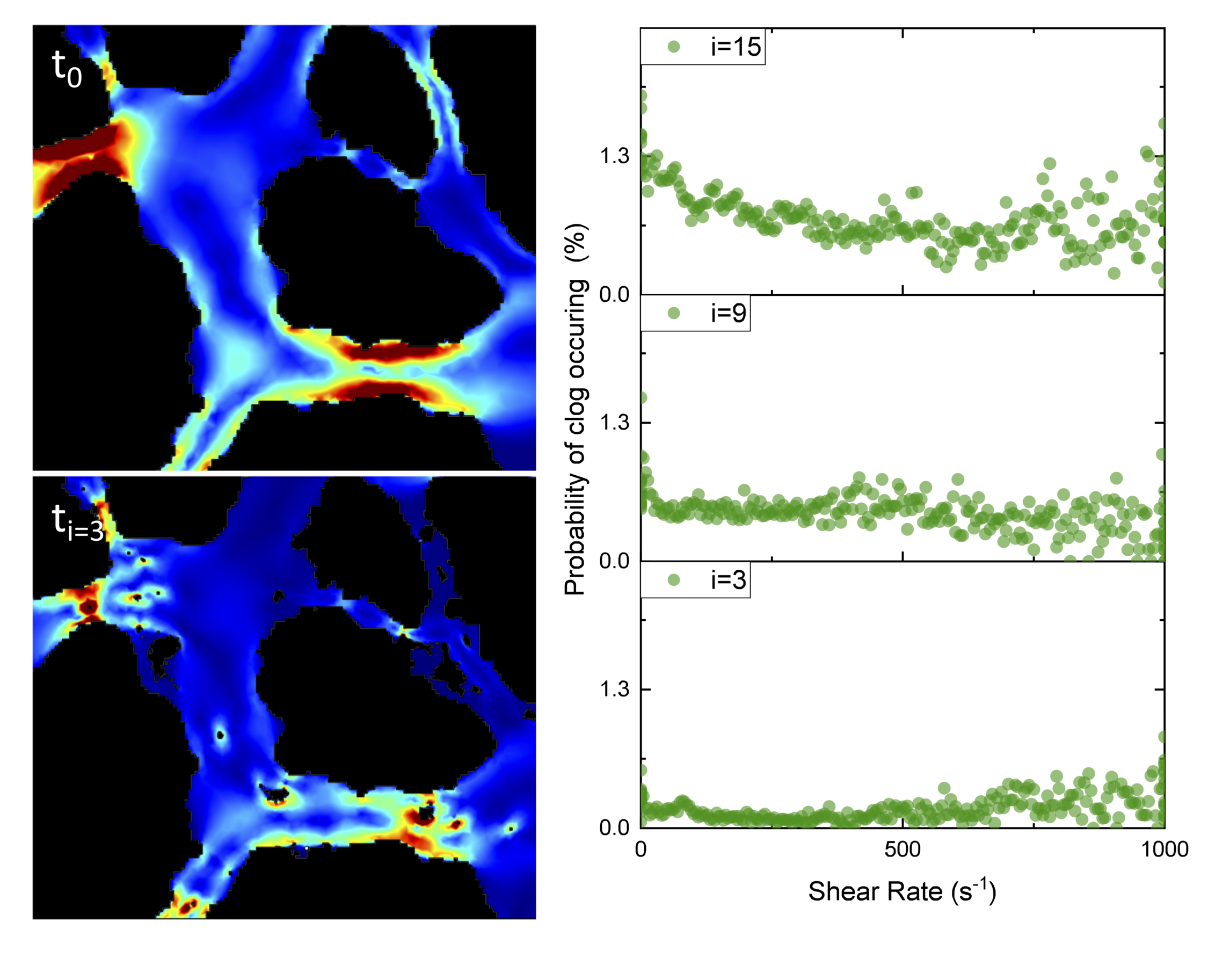

Supplement: S2 Fig — (left) An example of the simulated shear rate before (t0) and after subtracting the biofilm from the geometry (ti = 3) shows that biofilm influences pore space shear rates. (right) The relationship between shear rate and the probability of a clog occurring is relatively constant over time. (TIF) [file pone.0218316.s003.tif]

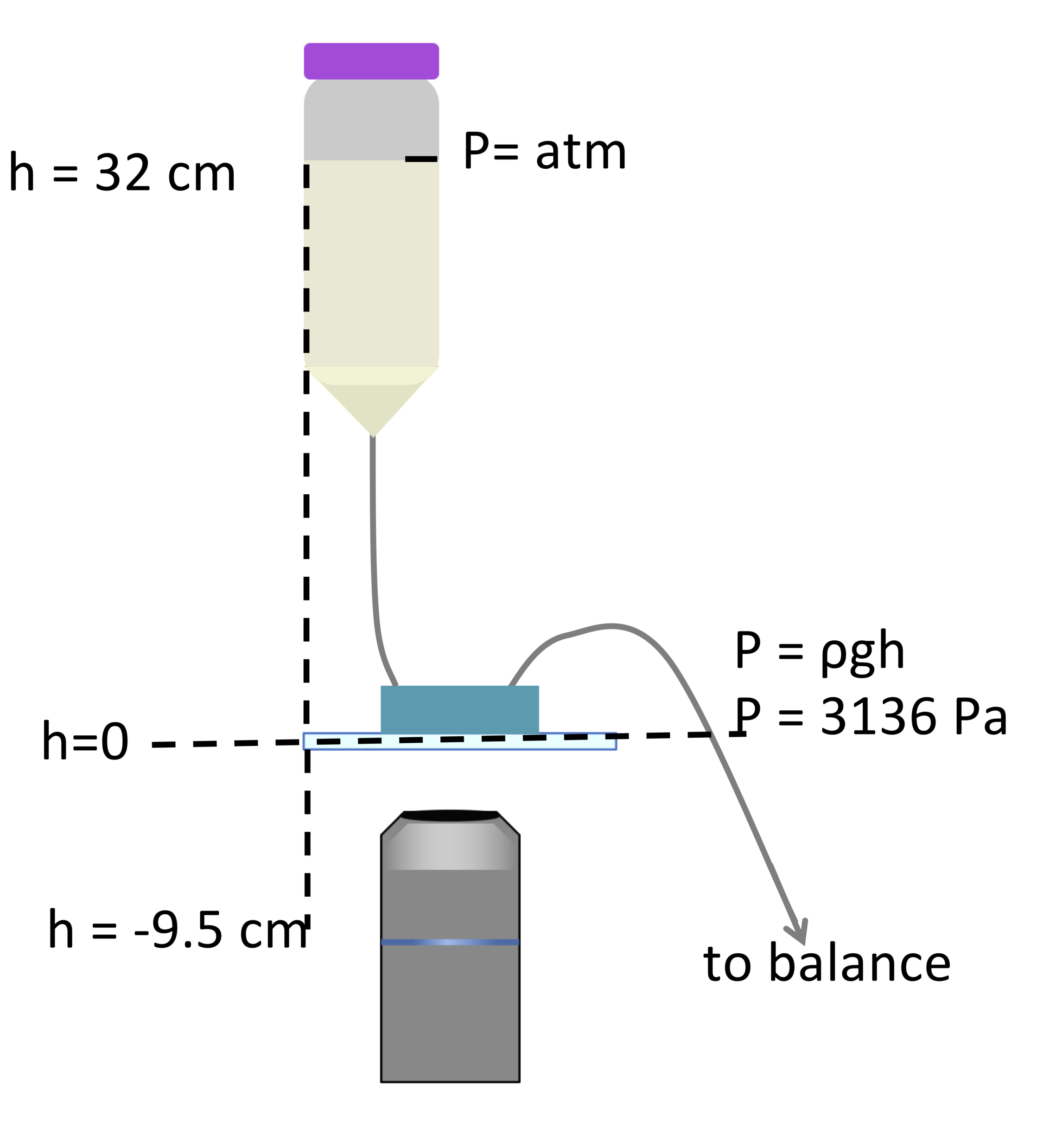

Supplement: S3 Fig — The lid on the reservoir tube was loosely fitted to assure atmospheric pressure above the liquid. (TIF) [file pone.0218316.s004.tif]

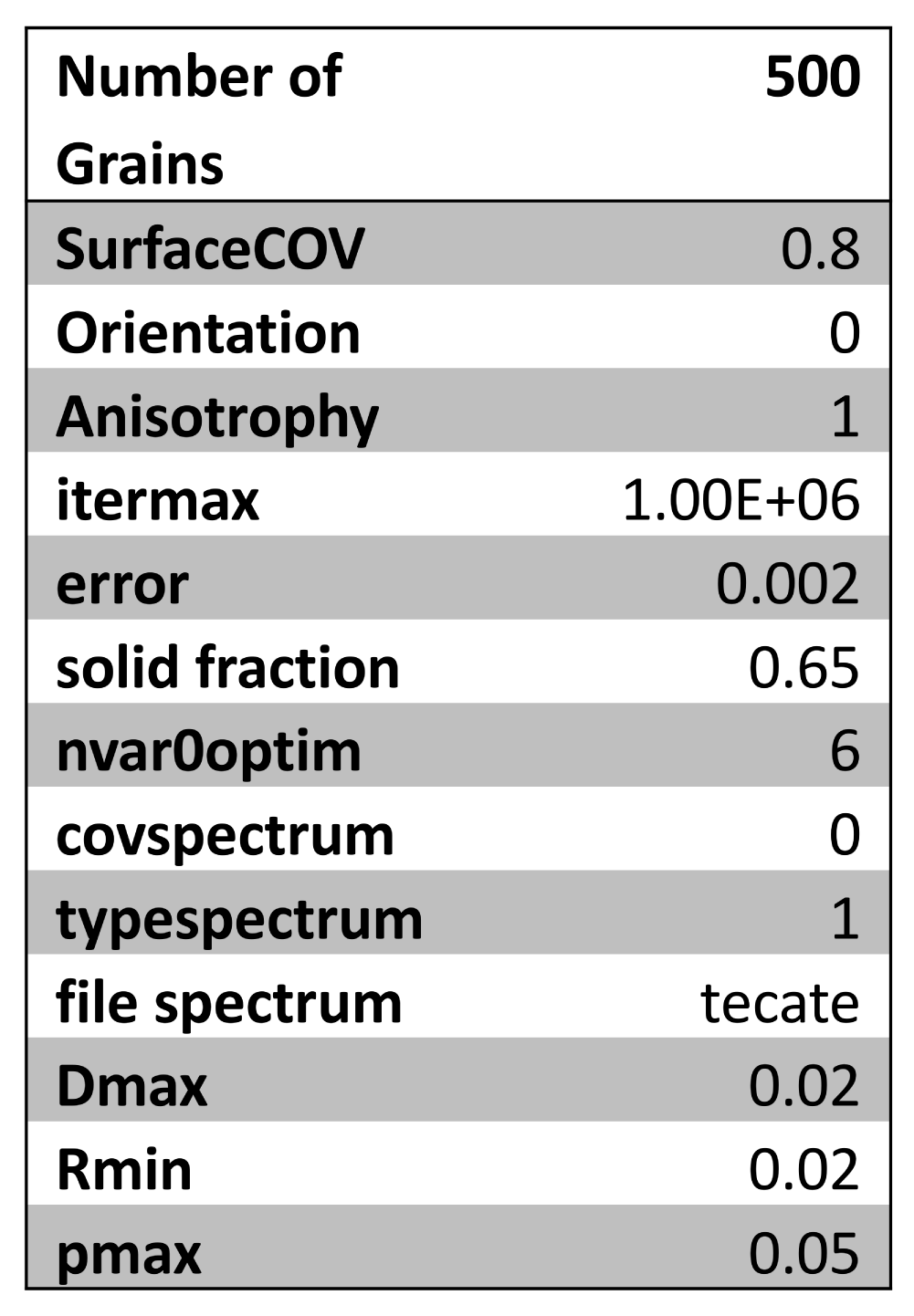

Supplement: S1 Table — (TIF) [file pone.0218316.s005.tif]
